# [Retracted] miR‑124 inhibits proliferation, migration and invasion of malignant melanoma cells via targeting versican

**DOI:** 10.3892/etm.2023.12346

**Published:** 2023-12-11

**Authors:** Ping Yang, Pingyuan Bu, Chengyuan Li

Exp Ther Med 14:3555-3562, 2017; 10.3892/etm.2017.4998

Following the publication of this paper, it was drawn to the Editors’ attention by a concerned reader that the Transwell assay data shown in Figs. 2D and 5E and the western blotting data shown in Fig. 3G were strikingly similar to data appearing in different form in other articles written by different authors at different research institutes that had either already been published elsewhere prior to the submission of this paper to *Experimental and Therapeutic Medicine*, or were under consideration for publication at around the same time. In view of the fact that certain of these data had already apparently been published previously, the Editor of *Experimental and Therapeutic Medicine* has decided that this paper should be retracted from the Journal. The authors were asked for an explanation to account for these concerns, but the Editorial Office did not receive a reply. The Editor apologizes to the readership for any inconvenience caused.

